# Conformations of the Pyranoid Sugars. II. Infrared Absorption Spectra of Some Aldopyranosides

**DOI:** 10.6028/jres.064A.025

**Published:** 1960-06-01

**Authors:** R. Stuart Tipson, Horace S. Isbell

## Abstract

The conformations of twenty-four aldopyranosides have been studied by analysis of their infrared absorption spectra. The most stable conformations of twelve of the glycosides had previously been assigned by Reeves from a study of their instability factors; these conformations were assumed to apply to the crystalline state, for which the spectra had been recorded. The compounds were classified into (a) configurationally and (b) structurally related groups, and the spectra were intercompared. The analysis revealed groups of absorption bands which showed a concerted shift on change of anomeric disposition.

With these groups of absorption bands thus identified, intercomparison with nine of the remaining spectra afforded evidence that the anomeric group (1) is axial in methyl d-*glycero*-*α*-l-*gluco*-heptopyranoside, methyl d-*glycero-α*-l-*manno*-heptopyranoside, and methyl d-*glycero-α*-d-*gulo*-heptopyranoside; (2) is equatorial in methyl 6-deoxy-*β*-l-mannopyranoside, methyl d-*glycero-β*-d-*gulo*-heptopyranoside, and cyclohexyl d-*glycero-β*-d-*gulo*-heptopyrnoside; and (3) either is quasi or occurs as different (or mixed) axial and equatorial forms in methyl *α*-d-lyxopranoside, methyl *β*-d-lyxopranoside, and (possibly) *α*-d-methylgulopyranoside.

Three of the glycosides were available as their crystalline complexes with calcium chloride. The spectra of these complexes were also examined, and the effect of co-crystallization with calcium chloride is pointed out.

## 1. Scope and Purpose of the Project

The shape or conformation of a molecule greatly influences its rates of reaction and other properties. For this reason, detailed knowledge of the conformations of pyranoid derivatives is desirable. Our prior publications on this subject have presented a system for naming the conformations of pyranoid compounds [l,2].^1^

The conformations of numerous methyl glycosides and other pyranoid derivatives have been determined by Reeves from (a) a study of stereomeric factors and (b) the type of complex formed in cuprammonia solution. Reaction of cuprammonia with an aldopyranoside may sometimes cause alteration in the conformation. Hence, assignments of conformation based on the formation of copper complexes need confirmation by measurement of at least one relevant physical characteristic.

The work herein reported was primarily undertaken to provide infrared spectrograms of aldosides having the pyranoid ring, with the object of discovering correlations that might be of value in conformational analysis. It seemed possible that the axial or equatorial disposition of reference groups in the various molecules of glycopyranosides should give rise to different vibrations, and that it should, accordingly, be feasible to identify certain bands in their spectra as being characteristic of the different ways of arranging the reference groups. The infrared spectra for a group of aldopyranosides have, therefore, been recorded, and the bands have been compiled and then studied by statistical and comparative methods.

Prior publications from our laboratory on related topics have dealt with the infrared absorption spectra of sugar acetates [3] and of some cyclic acetals of sugars [4], and with a system for classifying carbohydrate derivatives for comparative purposes [5]. The acetals previously studied are polycyclic and have fused or bridged rings; the conformations of such molecules are “locked.” On the other hand, the pyranoid ring of some unsubstituted glycopyranosides is flexible, and the conformation adopted may depend on the physical conditions; thus, the conformation of the molecules in solution, particularly in the presence of a complexing agent, may be different from that of the molecules in the crystalline material. In the present study, the infrared absorption spectra of compounds *in the solid phase* have been recorded, and a method has been developed for assignment of conformation from analysis of the infrared absorption spectra and comparison with the spectra of glycosides of known conformation.^2^

## 2. Compounds Investigated

Table 1 gives a list of the compounds, their code numbers [5],^3^ predicted stable conformations, and an index to the spectrograms; the serial number of a compound is the same as the number of its spectrogram. The spectra were measured in the region of 5000 to 667 cm^−1^ (sodium chloride optics) and in the region of 667 to 250 cm^−1^ (cesium bromide optics). The spectrograms are given together with a discussion of (a) the structure of the compounds and (b) some of the outstanding features of their spectra.

All of the compounds listed in table 1 are glycosides of aldoses, and all have the pyranoid ring. As a common structural feature, all but one of the glycosides have a glycosidic methoxyl group; one has a glycosidic cyclohexyloxy group. The glycosides differ in regard to one or more of the following features: (a) the *α* or *β* anomeric configuration at carbon atom 1, (b) the configurations of the other carbon atoms of the pyranoid ring, (c) the nature and configuration of the substituent, if any, at carbon atom 5 of the pyranoid ring, and (d) the configuration of carbon atom 5 in those glycosides in which this atom is asymmetric.

**Figure 1:**
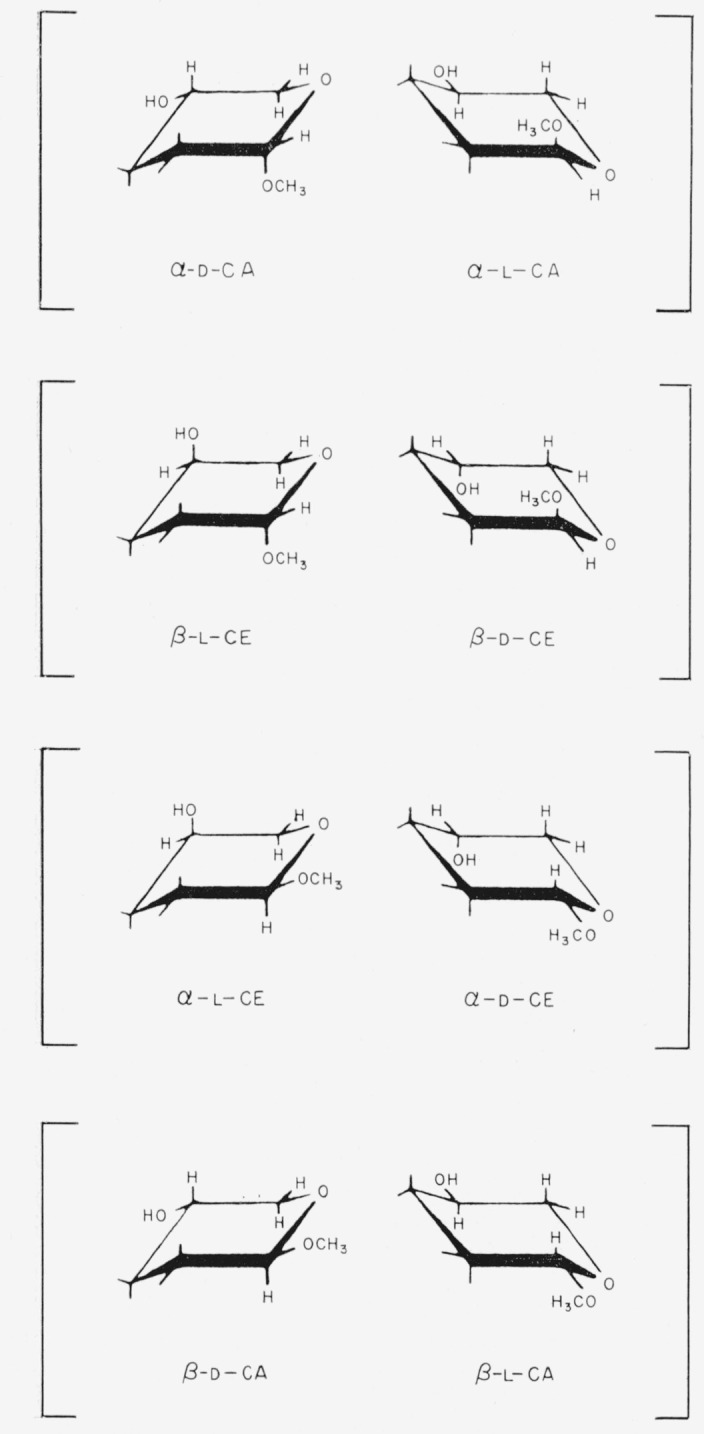
The four general chair-forms of the anomeric methyl aldopentopyranosides, and the enantiomorph of each. (The configurations at C2 and C3 are omitted.)

**Figure 2:**
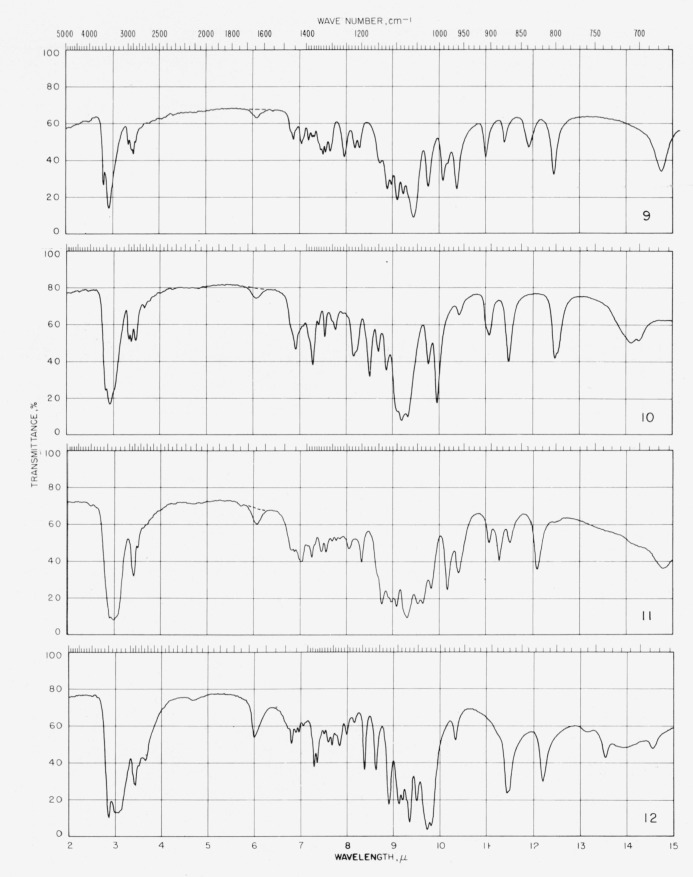
The four general chair-forms of the anomeric methyl 6-deoxyaldohexopyranosides (*R* = *CH_3_*), methyl aldohexopyranosides (*R* = *CH_2_OH*), and methyl aldoheptopyranosides (*R* = *CHOH–CH_2_OH*), with the enantiomorph of each. (The configurations at C2, C3, and C4 (and at C6, if asymmetric) are omitted.)

The conformation selected by Reeves as probably the most stable is shown in table 1 for each of those glycosides mentioned that he studied. The conformations are indicated by the system devised by Isbell and Tipson [1, 2]; the symbol CA means “that chair conformation for which the *α* anomeric group is axial,” and the symbol CE means “that chair conformation for which the *α* anomeric group is equatorial.” The chair forms and nomenclature for the anomers of the methyl aldopyranosides are shown in figures 1 and 2. The infrared absorption spectra of the same polymorphic modification of two members of an enantiomorphic pair are indistinguishable. Hence, in the present study, the spectrum of whichever member was available was examined, and was considered to apply to the other enantiomorph.

## 3. Reference Aldopyranosides of Known Conformation

Each methyl aldopyranoside is theoretically capable of assuming at least one of a variety of different conformations, depending on the conditions. From studies made by Reeves [6 to 8], it would appear that two groups (A and B) of methyl aldopyranosides can be distinguished. Glycosides in group A have one of the two chair-forms. The members of group B exist either (a) as a mixture of the two chair-forms or (b) as some other conformation.

It seemed likely that, in the solid state, each individual aldopyranoside would exist in only one conformation; and this conformation would presumably be the one it took as it crystallized from solution. As a working hypothesis, it was assumed that the conformation of an aldopyranoside in the crystalline state is the same as the stable form predicted by Reeves. It would then be reasonable to expect that examination of the infrared absorption spectra of the solid phase of those glycopyranosides of group A (each believed to assume a *single* chair-conformation), followed by a comparison with the spectra of the *other* group (B) of aldopyranosides, might provide evidence regarding the conformations (in the solid phase) of the members of the latter group (B).

For compounds 1, 2, 3, 4, 8, 9, 20, 21, 22, 23, and 24 (see table 1), the conformation predicted for each (by Reeves) as the most stable of all possibilities was found by him to be that actually adopted in cuprammonia; these glycosides comprise group A. Compounds 6, 7, and 12 form group B. For the anomers of methyl d-lyxopyranoside (compounds 6 and 7), Reeves at first predicted approximately equal stability for the CA and CE conformation of each, and his experimental results were in accord with the possibility of a mixture of the two conformations in cuprammonia; but he later suggested [9] that each might actually adopt one of the conformations in the boat-skew cycle. As regards compound 12 (methyl *α*-d-gulopyranoside), Reeves predicted approximately equal stabilities for the CA and CE conformations, but his experimental results indicated that the CA conformation is adopted in cuprammonia.

In addition to the spectra of these 14 glycosides studied by Reeves, those of 10 aldopyranosides of hitherto undetermined conformation have been recorded and analyzed; conformations have now been assigned to 7 of these glycosides. As regards compound 14 (methyl *β*-d-gulopyranoside), Reeves predicted that the CA conformation would be the most stable of all conformations, but he did not examine the behavior of this glycoside in cuprammonia.

Barker and Shaw [10] have devised a way of predicting the stable chair-conformation of each pyranose, which involves the assumption “that the degree of distortion in a molecule is determined by the total amount of overlap of non-bonded atoms, overlap between each pair being calculated separately and added together.” However, whereas a single, large overlap of *x* units might prohibit the adoption of a certain ideal conformation, a number of small overlaps (whose sum is equal to, or greater than, *x* units) might be accommodated by very slight departure of the conformation from ideality, so that, from the practical standpoint, the conformation in question is only slightly distorted. For this reason, the stable chair-conformations predicted by Reeves [6 to 8] have been used in the present study.

## 4. Classification of the Glycosides into Configurationally Related Groups

The 24 compounds were classified into three groups; the members of each group have like configurational features.

### 4.1. Aldopyranosides of the *xylo* Configuration

The members of this group of methyl aldopyranosides have the following general formulas (I) for the two chair-conformations.

**Figure:**
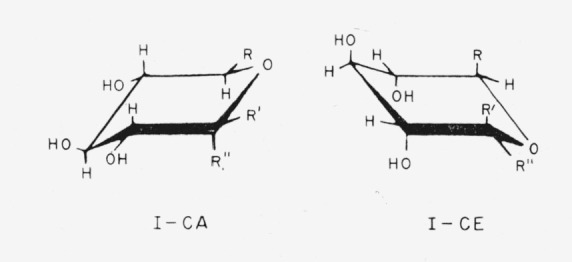


Compounds 1 to 5 presumably have one of the above general structures, with the following substituents.

1. Methyl *α*-d-xylopyranoside, R = H; R′ = H; and R″ = OCH_3_.
2. Methyl *β*-d-xylopyranoside, R = H; R′ = OCH_3_; and R″ = H.
3. Methyl *α*-d-glucopyranoside, R = CH_2_OH; R′ = H; and R″ = OCH_3_.
4. Methyl *β*-d-glucopyranoside, R = CH_2_OH; R′ = OCH_3_; and R″ = H.
5. Methyl d-*glycero α*-l-*gluco*-heptopyranoside (originally called “methyl *α*-d-*β*-galaheptopyranoside”),
  R is 
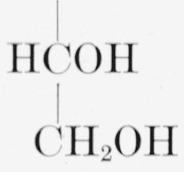
; R′ = H; R″ = OCH_3_; and the molecule is the mirror image of that depicted.

The following names (which have no official status) may be applied to compounds 3 to 5.

3. Methyl d-*glycero-α*-d-*xylo*-hexopyranoside.
4. Methyl d-*glycero-β*-d-*xylo*-hexopyranoside.
5. Methyl d-*threo*-*α*-l-*xylo*-heptopyranoside.

It should be noted that, in the CA conformation of compounds 2 and 4, all reference groups are equatorial; in the CE conformation of these compounds, all reference groups are axial.

### 4.2. Aldopyranosides of the *lyxo* Configuration

Three of the members of this group of configurationally related methyl aldopyranosides have the d-*lyxo* or d-*manno* configuration and the following general formulas (II) for the two chair-conformations.

**Figure:**
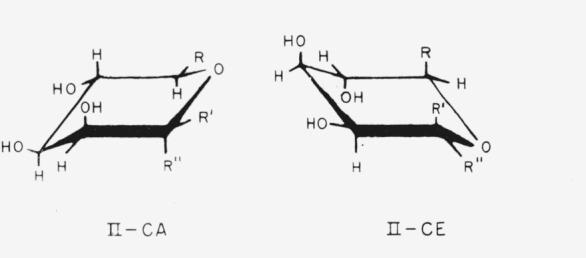


Compounds 6 to 8, when in one of the chair conformations, have one of the above general structures, with the following substituents.

6. Methyl *α*-d-lyxopyranoside, R = H; R′ = H; and R″ = OCH_3_.
7. Methyl *β*-d-lyxopyranoside, R = H; R′ = OCH_3_; and R″ = H.
8. Methyl *α*-d-mannopyranoside, R = CH_2_OH; R′ = H; and R″ = OCH_3_.

(Purely for comparative purposes, compound 8 can be regarded as methyl d-*glycero-α*-d-*lyxo*-hexopymnoside; the name has no official status.)

Compounds 9 to 11 have the l-*manno* configuration; the following general formulas (III) depict the two chair-conformations, which are essentially the mirror images of formulas II.

**Figure:**
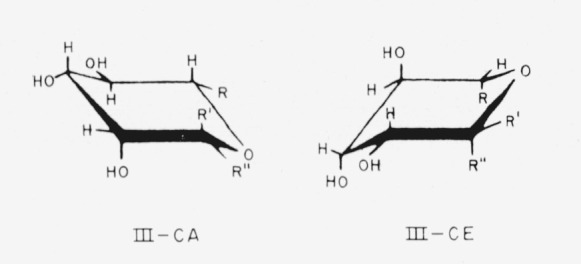


When existing in one of the chair conformations, compounds 9 to 11 have one of the above general structures, with the following substituents.

9. Methyl 6-deoxy-*α*-l-mannopyranoside (methyl *α*-l-rhamnopyranoside), R = CH_3_; R′ = OCH_3_; and R″ = H.
10. Methyl 6-deoxy-*β*-l-mannopyranoside (methyl *β*-l-rhamnopyranoside), R = CH_3_; R′ = H; and R″ = OCH_3_.
11. Methyl d-*glycero*-*α*-l-*manno*-heptopyranoside (originally called “methyl *α*-d-*α*-galaheptopyranoside”),
  R is 
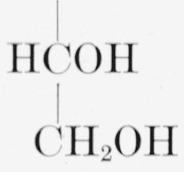
; R′ = OCH_3_; and R″ = H.

To emphasize the configurational relationships, the following unofficial names may be temporarily applied:

9. Methyl 6-deoxy-l-*glycero*-*α*-l-*lyxo*-hexopyranoside.
10. Methyl 6-deoxy-l-*glycero*-*β*-l-*lyxo*-hexopyranoside.
11. Methyl d-*threo-α*-l-*lyxo*-heptopyranoside.

Compounds 12 to 19 have the d-*gulo* configuration; the following general formulas (IV) depict their chair conformations, which are closely related to formulas III.

**Figure:**
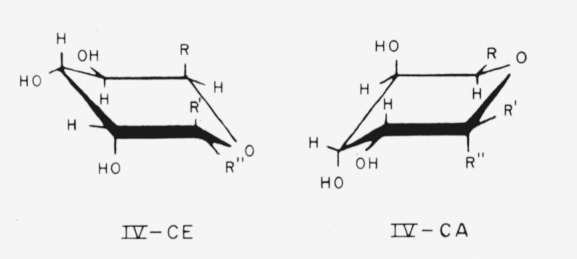

12. Methyl *α*-d-gulopyranoside monohydrate, R = CH_2_OH; R′ = H; and R″ = OCH_3_.
14. Methyl *β*-d-gulopyranoside, R = CH_2_OH; R′ = OCH_3_; and R″ = H.
15. Methyl d-*glycero-α*-d-*gulo*-heptopyranoside (originally called “methyl *α*-d-*α*-glucoheptopyranoside”),
  R is 
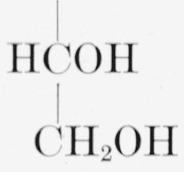
; R′ = H; and R″ = OCH_3_.
17. Methyl d-*glycero*-*β*-d-*gulo*-heptopyranoside (originally called “methyl *β*-d-*α*-glucoheptopyranoside”),
  R is 
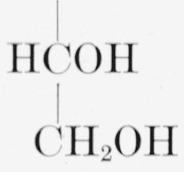
; R′ = OCH_3_; and R″ = H.
19. Cyclohexyl d-*glycero-β*-d-*gulo*-heptopyranoside,
  R is 
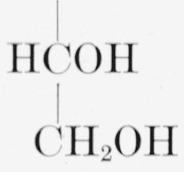
; R′ = OC_6_H_11_; R″ = H.

For comparison with compounds 6 to 8, and 9 to 11, the following unofficial names may be temporarily applied:

12. Methyl d-*glycero-β*-l-*lyxo*-hexopyranoside, monohydrate.
14. Methyl d-*glycero-α*-l-*lyxo*-hexopyranoside.
15. Methyl d-*erythro*-*β*-l-*lyxo*-heptopyranoside.
17. Methyl d-*erythro-α*-l-*lyxo*-heptopyranoside.
19. Cyclohexyl d-*erythro-α*-l-*lyxo*-heptopyranoside.

Because the *α*-*β* names are relative, not absolute, it will be noted that, *whenever C4 and C5 of a sugar have opposite configurations*, the CA conformation for the sugar when named as a substituted l-aldopentopyranose is the CE conformation when it is named as a d-aldohexopyranose.

### 4.3. Aldopyranosides of the *arabino* Configuration

This group of configurationally related glycosides consists of the methyl l-arabinopyranosides, the methyl d-galactopyranosides, and methyl 6-deoxy-*α*-l-galactopyranoside. The relationship of the l-arabinopyranosides to the d-galactopyranosides is the same as that of the l-lyxopyranosides to the d-gulopyranosides.

Compounds 20 to 23 may have one of the following structures (V and VI), with the indicated substituents.

**Figure:**
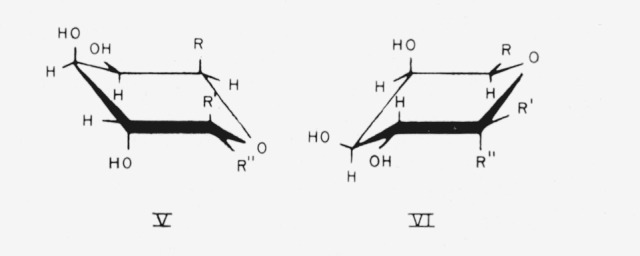

20. Methyl *α*-l-arabinopyranoside, R = H; R′ = OCH_3_; and R″ = H.
21. Methyl *β*-l-arabinopyranoside, R = H; R′ = H; and R″ = OCH_3_.
22. Methyl *α*-d-galactopyranoside, monohydrate, R = CH_2_OH; R′ = H; and R″ = OCH_3_.
23. Methyl *β*-d-galactopyranoside, R = CH_2_OH; R′ = OCH_3_; and R″ = H.

For purposes of comparison, compounds 22 and 23 may be temporarily given the following unofficial names:

22. Methyl d-*glycero-β*-l-*arabino*-hexopyranoside, monohydrate.
23. Methyl d-*glycero-α*-l-*arabino*-hexopyranoside.

Named in this way, the CE conformation is the same as the conformation that is called CA when they are named as d-aldohexopyranosides.

For compounds 20 and 21, formula V represents the CA conformation and formula VI represents the CE conformation. For compounds 22 and 23, named as d-aldoliexopyranosides, formula V represents the CE conformation and formula VI represents the CA conformation.

The possible chair conformations of compound 24 are the mirror images of those depicted above, with the following substituents.

24. Methyl 6-deoxy-*α*-l-galactopyranoside (methyl *α*-l-fucopyranoside), R = CH_3_; R′ = QCH_3_; and R″ = H.

The compound may, for purposes of comparison, be unofficially called methyl 6-deoxy-l-*glycero*-*β*-d-*arabino*-hexopyranoside. Named in this way, the conformation V having an axial (*β*-d) anomeric group is classified as CE; named as an *α*-l-hexopyranoside, it is classified as CA.

## 5. Discussion of the Spectra

In the present investigation, the *positions* of the various absorption bands for each of 24 aldopyranosides have been determined. For reasons discussed later, the relative intensities of absorption were not examined in detail.

The predicted stable conformations of 14 of these glycosides [6 to 8] are listed in table 1; eleven of these were accepted, but three (for compounds 6, 7, and 12) seemed open to question. In order that the conformation of these three and of each of the 10 remaining glycosides might be determined, the problem then resolved itself into a search of the infrared absorption spectra of the 11 “known” glycosides for potentially distinctive bands, followed by attempts to correlate the positions of these bands with such conformational and structural features as axial or equatorial disposition of (a) the glycosidic group at C1 or (b) the reference group (if any) at C5. The resultant findings were then applied in a study of the spectra of the glycosides of unknown conformation, in order to assign a conformation to each (in the solid state).

The spectra were examined in the groups outlined in table 2. However, before proceeding to a detailed analysis (see sections 5.3 and 5.4), some preliminary studies were made (in order to determine which methods of approach were likely to be the most fruitful).

### 5.1. Effect of Calcium Chloride of Crystallization

Glycosides 12, 15, and 17 lacked the following bands shown by their compounds with calcium chloride (group 1 of table 2): 3226 to 3215 cm^−1^; 1745 cm^−1^; 1366 to 1364 cm^−1^; 1148 to 1130 cm^−1^; 1117 to 1114 cm^−1^; 1049 to 1044 cm^−1^; 772 to 771 cm^−1^; and 655 to 643 cm^−1^. Because co-crystallization with calcium chloride alters the spectrum, either by removal or displacement of certain bands, only the spectra of *the 21 compounds lacking calcium chloride* were intercompared with respect to the other structural, configurational, and conformational features (groups 2 to 14 of table 2).

### 5.2. Preliminary Evaluation of Configurational and Constitutional Effects

Kuhn [11] recorded the spectra (in the range of 1250 to 667 cm^−1^) for compounds 2, 3, 4, 8, 22, and 23. He noted that “the difference between the anomeric forms shows up very nicely.” In table 1 are listed eight anomeric pairs, namely 1, 2; 3, 4; 6, 7; 9, 10; 12, 14; 15, 17; 20, 21; and 22, 23. A cursory inspection of their spectra revealed that Kuhn’s remark applies to all of these pairs.

When the two spectra for an anomeric pair are compared, it is seen that (a) they have certain bands “in common” and (b) each shows certain bands that are “absent” from the other. With the information at hand, it was not known whether the absence of a band in one spectrum (and its presence in the other spectrum) is real or is actually due to a shift to some other position. If the “absent” band has actually been shifted to a position matching a band in the other spectrum, it will temporarily be regarded as being a band “common to the two spectra.” On the other hand, if a band is shifted to a position not matched in the other spectrum, it will be observed as being “present” as a distinguishing band. Hence, some bands that may actually be ascribable to *different* features may be paired for the two spectra, and some bands (in the two spectra) that are actually ascribable to the *same* feature may appear to differentiate between the two.

Nevertheless, if the bands “differentiating” the two members of *one* anomeric pair are found to bear some relationship to the bands differentiating the two members of a *different* anomeric pair, it is reasonable to ascribe this to some influence that is operative in both instances.

For molecules as complex as those of the aldopyranosides, many of the observed bands cannot yet be assigned to particular vibrational modes. Assignments for some of the bands are given in section 5.5. However, we are not here concerned with (a) which bands, arising from vibrations localized in a functional group, are relatively independent of the remainder of the molecule, or (b) which bands involve other parts of the molecule (and are, therefore, sometimes perturbed in unexpected ways when the molecule is altered). Instead, in the present treatment, a band in two different spectra is regarded as “a band common to the two spectra” if it occupies approximately the same *position* in the spectrum, regardless of (a) whether it is actually contributed by vibrations which are of the same nature in the two molecules, or (b) the relative intensity in the two spectra. In our analysis, all of these bands are given equal weight.

Before proceeding to a detailed study, an examination of effects possibly attributable to configuration and constitution was undertaken. Simultaneously, an attempt was made to ascertain whether any of the differentiating bands could be regarded as being indicative of the axial or equatorial disposition of the anomeric group. It was realized that, if the anomeric group of an *α* anomer is axial, the conclusion that the anomeric group of its *β* anomer is equatorial might not necessarily follow—the disposition might, for example, be quasi; and *vice versa.* Therefore, to avoid unconscious bias that might arise from the use of the customary *α* or *β* names, only the assigned serial numbers of the above eight pairs of glycosides were used when making intercomparisons of their spectral characteristics.

Those of our compounds whose stable conformations had been assigned by Reeves [6 to 8] were all *methyl* glycosides. Consequently, from the total of 24 aldopyranosides listed in table 1, the cyclohexyl glycoside (compound 19) was excluded in this part of the study. Moreover, in view of the effects noted in section 5.1, the three compounds containing calcium chloride of crystallization (group 1 of table 2) were also excluded.

The generalized similarities and differences in the methyl aldopyranosides studied may be summarized as follows.

**Table:** 

C1	C2 C3 C4	C5
		
Disposition of OCH_3_	Group-configuration	Substituent
		
*a*	*xylo*	H
or *e*	or *lyxo*	or CH_3_
or *q*	or *arabino*	or CH_2_OH or CHOH-CH_2_OH

No glycoside having the *ribo* group-configuration is included in this investigation.

#### a. Effect of Change of Group-Configuration on Positions of Bands Common to Spectra (for Each Group-Configuration)

By group-configuration is meant the configuration of the glycoside, regardless of its anomeric form (see groups 2, 3, and 4 of table 2). In this analysis, the infrared spectra were examined for eight anomeric pairs (see above). The bands common to any one pair of anomers were systematically compared with the bands common to each and every other pair of configurationally related anomers. The three pairs of methyl pentopyranosides available for study were not configurationally related; each pair of pentopyranosides was compared with the related 5-*C*-substituted derivatives. For any one anomeric pair of pentopyranosides, these comprised anomeric pairs (and a few single anomers) of one or more of the following derivatives: 5-*C*-methyl, or methyl 6-deoxyaldohexopyranosides; 5-*C*-(hydroxymethyl), or methyl aldohexopyranosides; and 5-*C*-(1,2-dihydroxyethyl), or methyl aldoheptopyranosides. Three such configurational groups were available for study, namely, groups 2, 3, and 4 of table 2. Essentially the same kind of procedure was adopted in examining each group, and so a description of the method used will be exemplified by discussion of its application to group 2.

For group 2, the bands *differentiating* the *α* from the *β* anomer of methyl l-arabinopyranoside were tabulated and set aside for later evaluation (see section 5.3), and those bands *common* to the two anomers were tabulated. Similarly, the bands differentiating the *α* from the *β* anomer of methyl d-galactopyranoside were tabulated and set aside (see section 5.4), and the bands *common* to these two anomers were tabulated. It was then observed that *introduction of the 5-C-(hydroxymethyl) group in the pentopyranoside*, to afford the hexopyranoside, *had resulted in the display of a different spectrum of bands.* (The fact that the group-configuration was enantiomorphic may be ignored in this connection.) This observation is further developed in section 5.2.c.

In view of this effect of substitution at C5 of the pentopyranoside, a further winnowing of bands was undertaken. Those bands differentiating the pentopyranosides from the hexopyranosides were set aside for later consideration (see sections 5.3 and 5.4), and the bands *common* to the two pentopyranosides *and* the two hexopyranosides were tabulated. Finally, such of these bands as were *also* shown by the sole 6-deoxyaldohexopyranoside in this group (namely, compound 24) were selected and tabulated. This afforded a table recording the bands shown by all the glycosides (in this study) that have the *arabino* or *galacto* configurations.

In a similar manner, a table was compiled of the bands shown by all the members of group 3 (of table 2), and another table of the bands shown by all the members of group 4. These tables recorded, for each group-configuration, the bands that are shown *regardless* of anomeric disposition or of substitution (or nonsubstitution) at C5.

Inspection of these three tables revealed that *a change in group-configuration results in changes in the positions of a number of bands*, common to one configurational group, relative to those common to another configurational group. As a corollary, a set of bands shown by one group-configuration may tentatively be regarded as characteristic of that group-configuration.

Incidentally, on intercomparing the three groups, it was noted that the 21 glycosides comprising groups 2, 3, and 4 have, after application of the winnowing described, only four bands in common, namely, those at 2882 to 2841 cm^−1^, 1368 to 1330 cm^−1^, 1153 to 1111 cm^−1^, and 1109 to 1087 cm^−1^ (see sec. 5.5). These bands are displayed regardless of the anomeric disposition or of substitution (or nonsubstitution) at C5.

#### b. Effect of Change of Group-Configuration on Positions of Bands Which Differentiate Two Anomers

The bands that differentiate the two members of each anomeric pair were tabulated; they are listed and discussed in sections 5.3 and 5.4. Study of these tables revealed that, for the aldopentopyranosides, change from the *xylo* to the *arabino* configuration leaves the majority of the anomer-differentiating bands substantially *unchanged* in position (see sec. 5.3). For the 5-*C*-substituted aldopentopyranosides, change from one configuration to another (e.g., of the aldohexopyranosides) causes *changes* in the positions of bands that differentiate anomers.

#### c. Effect (on the Spectra) of Various Substitutions at Carbon Atom 5 of Aldopentopyranosides

In this analysis, bands shown by an anomeric pair of aldopentopyranosides (group 5 of table 2) were first compared with those shown by 5-*C*-substituted derivatives (groups 6, 7, and 8). It was found that substitution at C5 of the aldopentopyranosides caused shifts in the anomer-differentiating bands.

However, intercomparison of the spectra of the 5-*C*-substituted derivatives revealed that, for *any one group-configuration*, change in the substituent from the methyl to the hydroxymethyl or to the 1,2-dihydroxyethyl group *did not* cause profound shifts of anomer-differentiating bands.

To summarize the observations in sections 5.2a, b, and c, it is seen that, for the diagnostic purposes under consideration, the spectra of the 5-*C*-substituted aldopentopyranosides of any one group-configuration may be intercom pared, but they should not be intercompared with the spectra of their isomers having a different group-configuration, nor with those of the related aldopentopyranosides. On the other hand, intercomparison of the spectra of members of groups 6, 7, and 8 that have the *same* configuration might afford fruitful results.

### 5.3. Absorption Bands Possibly Indicative of the Axial or Equatorial Anomeric Group of the Methyl Aldopentopyranosides

As the starting point in this series of analyses of spectra, we selected methyl *β*-d-xylopyranoside (compound 2) because, if this compound adopts a chair conformation, the reference groups will either be all axial (CE) or all equatorial (CA). Its spectrum was compared with that of its *α* anomer (compound 1), in order to determine the effect (on the spectrum) of changing the anomeric group from equatorial to axial, or vice versa. A similar comparison was now made for the anomers of methyl l-arabinopyranoside (compounds 20 and 21).

Bands that are essentially the same for *both* anomers of (a) the methyl d-xylopyranosides or of (b) the methyl l-arabinopyranosides, or of (c) both configurational groups, are given^4^ in table 3. It seemed reasonable to assume tentatively that bands shown by *all* of these glycosides might be independent of total configuration, whereas those shown by one pair of anomers having the same group-configuration might be a reflection, via shifting of bands, of an effect of the total configuration of that pair.

In table 4 are given the bands shown by *one* anomer (but not the other) of the methyl d-xylopyranosides and the methyl l-arabinopyranosides. If these “anomer-differentiating” bands have any relationship to the axial or equatorial disposition of the respective glycosidic methoxyl group, the results in table 4 indicate that (a) compounds 2 and 20 have the same anomeric disposition, and (b) compounds 1 and 21 have the same anomeric disposition; that is, in both instances, both are equatorial or both are axial. If the conformation predicted by Reeves [7, 8] as being the most stable *for any one of these four compounds* is accepted for the crystalline state, the conformations of the other three may be deduced from the results in table 4. For example, if the anomeric group of methyl *β*-d-xylopyranoside is equatorial and that of its *α* anomer is axial, the results indicate that the anomeric group of methyl *α*-l-arabinopyranoside is equatorial and that that of its *β* anomer is axial. These conclusions are in complete agreement with Reeves’ assignments (see table 1). It may be noted that the sole difference between methyl *β*-d-xylopyranoside-CA and methyl *α*-l-arabinopyranoside-CE lies in the configuration of carbon atom 4.

**Figure:**
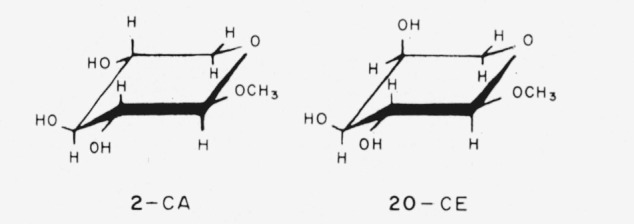


The corresponding bands of the methyl d-lyxopyranosides are also given in table 4. The results suggest that, for each anomer thereof, either (a) a conformation having a quasi or a different kind of axial or equatorial anomeric group is adopted in the crystalline state, or (b) a mixture of the CA and CE conformations crystallized together. These conclusions *also* agree with Reeves’ predictions (see table 1).

It should be noted that, in this series of intercomparisons, there was no possibility of band displacements attributable to the effect of a substituent at carbon atom 5. However, displacements (to lower or higher wavenumbers) caused by the differences in configuration might have been either (a) retained or (b) partially or wholly eliminated by the arbitrary procedure of temporarily ignoring bands that are essentially the same for both anomers of methyl d-xylopyranoside or of methyl l-arabinopyranoside, or both.

### 5.4. Analysis of the Spectra of Groups of Configurationally Related Aldopyranosides, Excluding the Aldopentopyranosides

In this analysis, two potential sources of misinterpretation were avoided. Firstly, intercomparisons were not made between the aldopentopyranosides and (a) the 6-deoxyaldohexopyranosides, (b) the aldohexopyranosides, and (c) the aldoheptopyranosides, because of the band displacements caused by the various substituents at C5 of the aldopentopyranosides. On the other hand, it was assumed that change from a methyl substituent at C5 to either a hydroxymethyl or a 1,2-dihydroxyethyl substituent would occasion no change in conformation; shifts of bands have already been discussed (see sec. 5.2). Secondly, intercomparisons were confined to intra-group study of groups of glycosides having *the same configuration* for each member of one group. In this way, band shifts attributable to change of configuration did not come into consideration.

First of all, as *a check on the significance* of the results accruing from our method of comparing absorption bands, the spectra of the anomers of methyl d-galactopyranoside (compounds 22 and 23) were compared with that of methyl 6-deoxy-*α*-l-galactopyranoside (compound 24). The results are given in table 5; column C gives bands shown by the *β* anomer but not by the two *α* anomers, and column D records bands shown by the two *α* anomers but not by the *β* anomer. It may be concluded that, assuming the validity of the correlations, if the anomeric group of the *α* anomers is axial, that of the *β* anomer is equatorial, or vice versa. If the conformation predicted by Reeves [7] as being the most stable for *any one of these three compounds* is accepted for the crystalline state, the deduced conformations for the other two are in complete agreement with Reeves’ assignments for them (see table 1).

The anomeric disposition of methyl *α*-d-gulopyranoside (compound 12) was now studied. Its spectrum was compared with those of methyl *α*-d-mannopyranoside (compound 8) and methyl *β*-d-gulopyranoside (compound 14). The results are given in table 6; column C records bands shown by compound 12 that are absent from the spectra of compounds 8 and 14; column D gives the bands common to compounds 8 and 12; and column E gives the bands common to compounds 8 and 14, not shown by compound 12. Thus, the spectrum of compound 12 has some resemblances to that of compound 8 and some to that of compound 14, but it also differs from both. Now, for the most stable conformation of compound 8, Reeves [8] predicted an axial anomeric group; for compound 14, he predicted [12] an equatorial anomeric group. The results in table 6 suggest that, if these assignments are accepted, compound 12 in the crystalline state either (a) assumes a conformation other than the chair form, possibly one having a quasi anomeric group, or (b) consists of a mixture of the CA and CE conformations. These conclusions agree with Reeves’ *prediction* for compound 12 (see table 1). It should be noted that compound 12 may be an example of a glycoside whose stable conformation is altered by cuprammonia, because, in this solvent, it adopts the CA conformation [8]. Alternatively, it is possible that, for the purpose under consideration, a sugar derivative having the *manno* configuration should not be compared with related derivatives having the *gulo* configuration.

All of the foregoing deductions are compatible with Reeves’ assignments. Consequently, the validity of the deductions (and the reliability of the method of analysis of the spectra) was apparently established. The spectra of the remaining glycosides (compounds 5, 10, 11, 15, 17, and 19) were, therefore, examined in order to assign an anomeric disposition to each. No prediction has been made as to the stable conformation for each of these glycosides.

A comparison of the spectrum of methyl d-*glycero-α*-l-*gluco*-heptopyranoside (compound 5) with those of the anomers of methyl d-glucopyranoside (compounds 3 and 4) indicated its resemblance to the spectrum of methyl *α*-d-glueopyranoside (compound 3); see column C of table 7. If Reeves’ prediction that the most stable conformation for compound 3 is the CA, this observation suggests that compound 5 has the CA conformation in the crystalline state.

The spectrum of methyl 6-deoxy-*β*-l-mannopyranoside (compound 10) was compared with those of its *α* anomer (compound 9) and of methyl *α*-d-mannopyranoside (compound 8). The results are given in table 8; column C records three bands shown by compounds 8 and 10 that are not shown by compound 9. However, column D (table 8) reveals that the two *α* anomers have some 13 bands in common that are not exhibited by compound 10. Since these two *α* anomers have the CA conformation as their predicted stable conformation [8], these results suggest that compound 10 has an equatorial anomeric group.

The spectrum of methyl d-*glycero-α*-l-*manno*-heptopyranoside (compound 11) was now compared with the spectra of compounds 8, 9, and 10. The results are given in table 9, except that, to avoid repetition of much of the information in table 8, the bands common to all four of these glycosides are omitted. Column A (table 9) records three bands shared by compound 11 and methyl 6-deoxy-*β*-l-mannopyranoside (compound 10); column B gives 17 bands that are shown by compound 11 and also by methyl *α*-d-mannopyranoside (compound 8) or methyl 6-deoxy-*α*-l-mannopyranoside (compound 9), or both. Since the predicted stable conformation for the latter two compounds is the CA conformation, this evidence indicates that the anomeric group of compound 11 is axial.

The spectra of the anomers of methyl d-*glycero*-d-*gulo*-heptopyranoside [compounds 15 (*α*) and 17 (*β*)] were compared with the spectrum of methyl *β*-d-gulopyranoside (compound 14). Compound 14 showed a band at 751 cm^−1^; in the same region, compound 15 showed bands at 764 and 738 cm^−1^. In contrast, compounds 14 and 17 share 17 bands not shown by compound 15 (see column C of table 10). If the CA conformation predicted as being the stable conformation for compound 14 is accepted, these results suggest that the anomeric group is equatorial in compound 17 and nonequatorial (that is, axial or quasi) in compound 15.

Finally, the spectrum of cyclohexyl d-*glycero-β*-d-*gulo*-heptopyranoside (compound 19) was studied in comparison with the spectra of compounds 15 and 17 (the *α* and *β* anomers of the corresponding methyl glycoside). To avoid repetition of much of the information in table 10, the bands common to all three of these glycosides are not given here. The spectrum of compound 15 lacked the following bands in the spectra of compound 17 and (in parentheses) of compound 19: 3509 (3484); 3448 (3436); 2732? (2653?); 1330 (1333); 1311 (1304); 1287 (1287); 1212 (1209); 1122 (1129); 1056 (1056); 1006 (1006); 801 (808); 599 (594); and 575 (575) cm^−1^. If the previous finding is accepted [namely, that the anomeric group of methyl d-*glycero-β*-d-*gulo*-heptopyranoside (compound 17) is equatorial, but that that of the *α* anomer, compound 15, is not], these results suggest that the anomeric group of cyclohexyl d-*glycero-β*-d-*gulo*-heptopyranoside is equatorial.

For reasons discussed in section 5.1, the spectra of compounds 13, 16, and 18 were not further examined.

### 5.5. Other Absorption Bands

All of the spectra were studied in regard to the other features listed in table 2. The hydrates (group 9) showed a band at 1664 to 1634 cm^−1^.

Compound 19, having a cyclohexyloxy group, showed bands at 2933, 2890, and 2857 cm^−1^, possibly characteristic of —CH_2_—(C—H stretching). It also showed hands at 1449 and 1441 cm^−1^, possibly attributable to —CH_2_—(C—H deformation).

All of the compounds showed at least one band in the region of 3413 to 3279 cm^−1^ (associated alcoholic —O—H stretching); at 3012 to 2915 cm^−1^ (C—H stretching); at 1466 to 1441 cm^−1^, and at 1346 to 1316 cm^−1^ (C—H bending); and at 1267 to 1235 cm^−1^ (C—O). Except for compound 15, all of the compounds showed at least one band in the region of 1247 to 1211 cm^−1^ (C—O stretching). Compounds 3, 6, 8, 13, and 20 showed a band at 3289 to 3279 cm^−1^ (H bonding?).

As previously mentioned, all of the methyl glycopyranosides (group 11 of table 2) show a band in the range of 2882 to 2841 cm^−1^. This may possibly be attributable to the glycosidic methoxyl group, because Henbest and coworkers [13] have observed that methoxyl groups absorb in the range of 2832 to 2819 cm^−1^. All of the methyl glycopyranosides also show bands at 1368 to 1330 cm^−1^, 1285 to 1245 cm^−1^, 1153 to 1111 cm^−1^, and 1109 to 1087 cm^−1^. A band near 1100 cm^−1^ is characteristic [14] of the methoxyl groups in methoxy-steroids.

## 6. Experimental Procedures

### 6.1. Preparation and Purification of the Compounds

The compounds listed in table 1 were prepared by the methods given in the references cited. Most of the compounds were prepared in the course of an earlier study on the configuration and conformation of methyl glycosides, with reference to optical rotations and rates of hydrolysis [15]. Each substance was recrystallized from an appropriate solvent until further recrystallization caused no change in its melting point or optical rotation.

### 6.2. Preparation of the Pellets

Samples for spectrophotometric study were prepared in the solid phase, as pellets of the crystalline glycoside suspended in an alkali-metal halide, exactly as previously described [4]. For the range of 5000 to 667 cm^−1^, a concentration of 0.4 mg of glycoside per 100 mg of potassium chloride was used. For the range of 667 to 250 cm^−1^, the following weights of glycoside per 100 mg of potassium iodide were used—compounds 1, 2, and 20: 1.33 mg; compound 5: (A) 0.33 and (B) 2 mg; compound 24: 3 mg; and for the rest of the compounds: 2 mg. In addition, for the range of 667 to 333 cm^−1^, the spectrum of compound 12 at a concentration of 2 mg per 100 mg of potassium *chloride* was recorded. Comparisons of intensity of absorption, from one compound to another, can only be true and quantitative where the molar concentration is the same.

### 6.3. Measurement of Infrared Absorption

**Figure 3:**
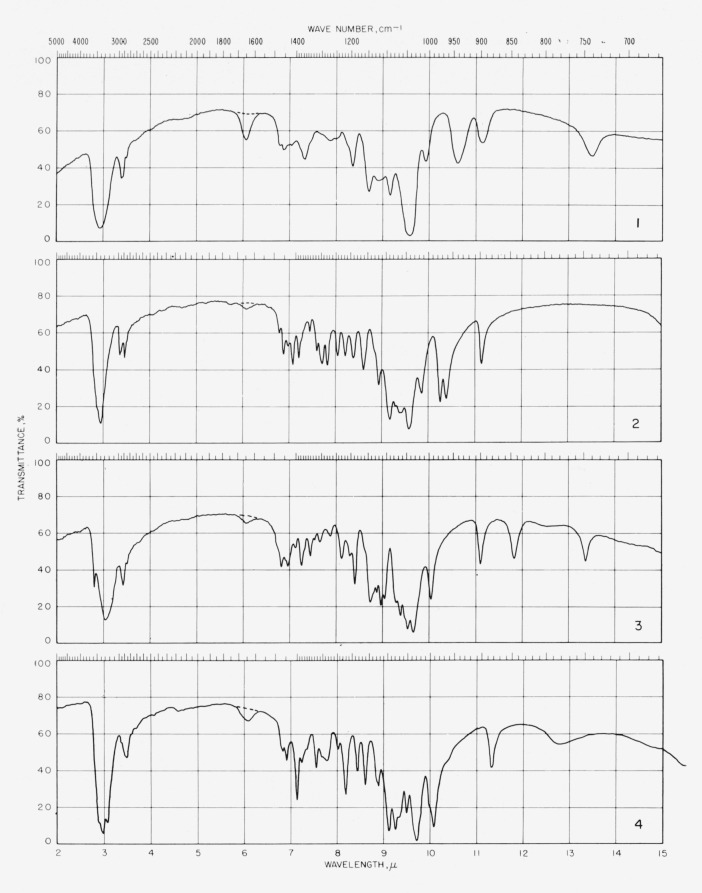

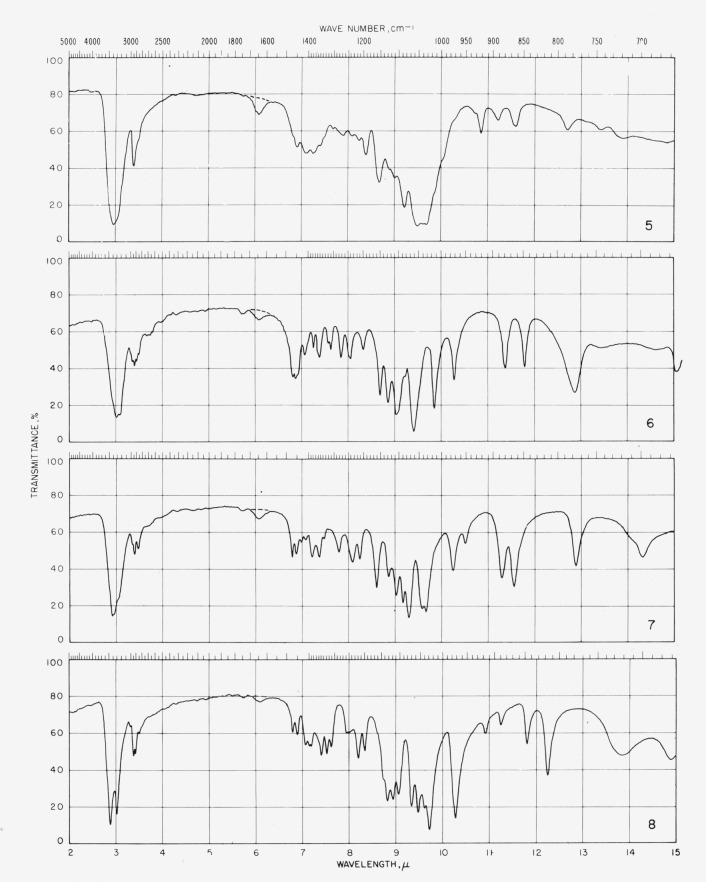

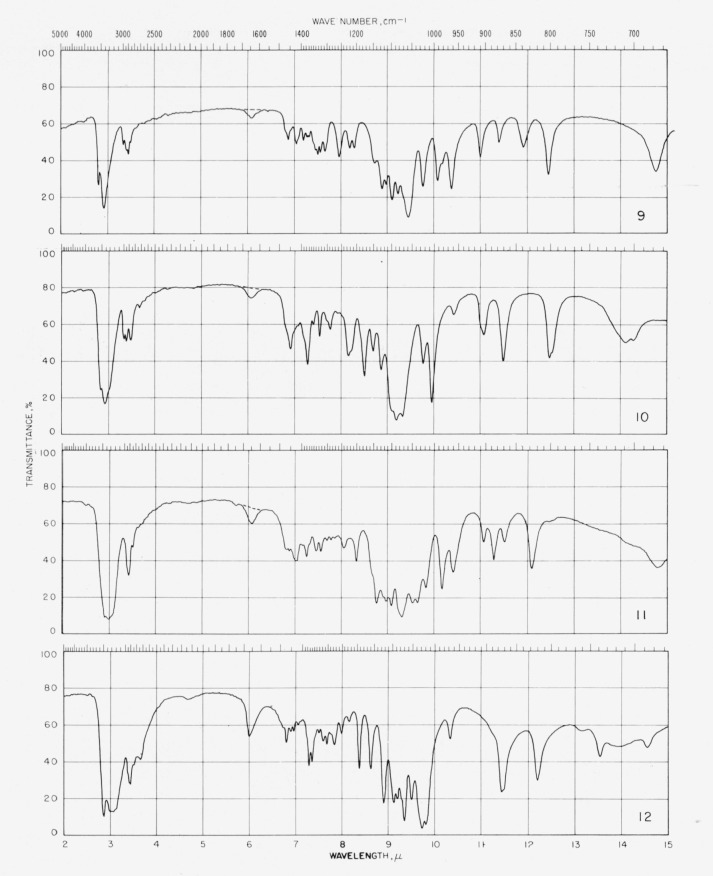

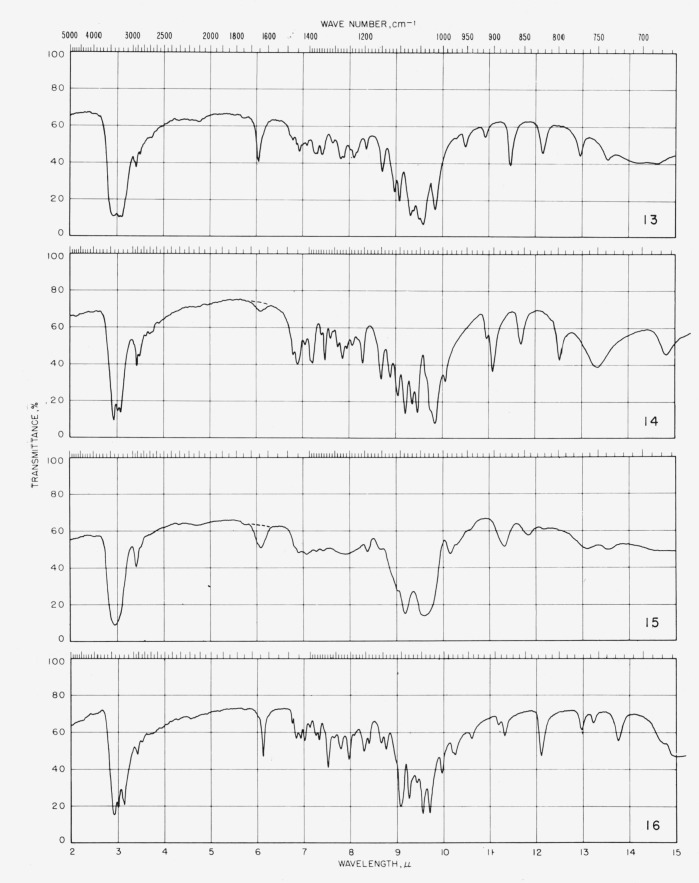

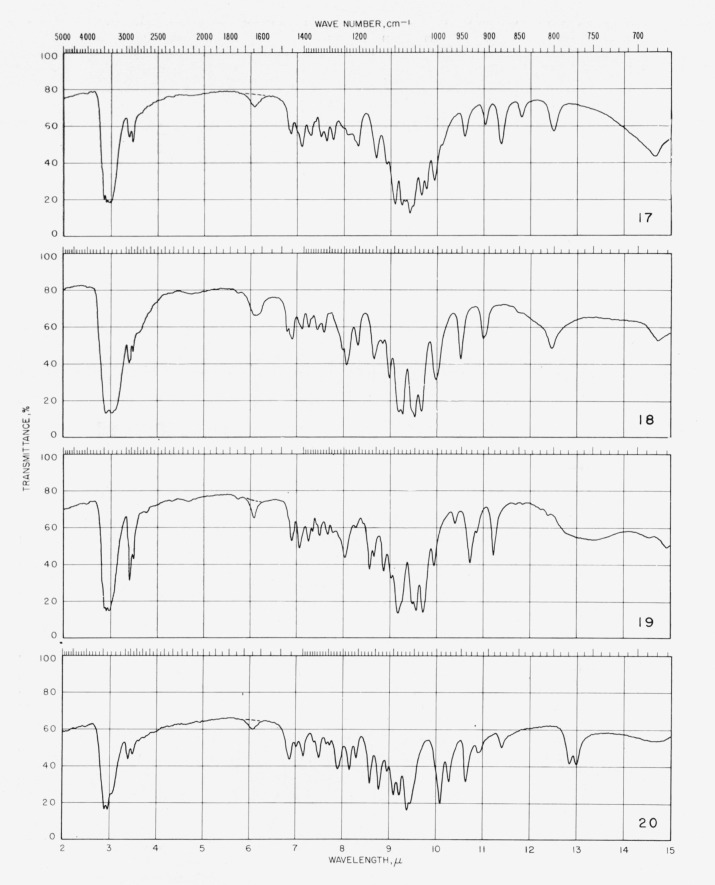

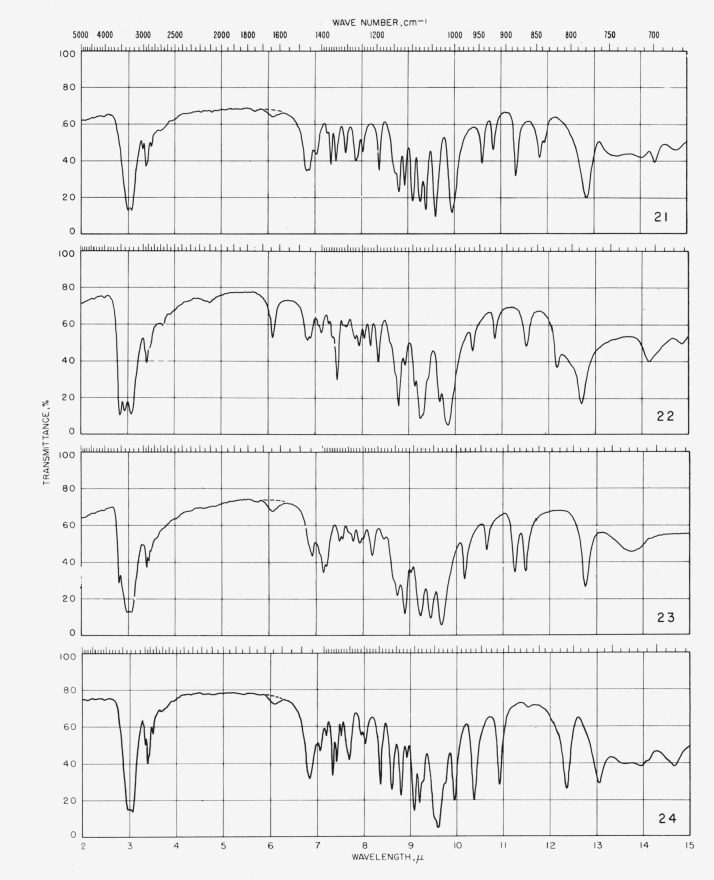
Spectrograms of materials in potassium chloride pellets. **1**, Methyl *α*-d-xylopyranoside; **2**, methyl *β*-d-xylopyranoside; **3**, methyl *α*-d-glucopyranoside; **4**, methyl *β*-d-glucopyranoside. **5**, methyl d-*glycero-α*-l-*gluco*-heptopyranoside; **6**, methyl *α*-d-lyxopyranoside; **7**, methyl *β*-d-lyxopyranoside; **8**, methyl *α*-d-mannopyranoside. **9**, methyl 6-deoxy-*α*-l-mannopyranoside; **10**, methyl 6-deoxy-*β*-l-mannopyranoside; **11**, methyl d-*glycero*-*α*-l-*manno*-heptopyranoside; **12**, methyl *α*-d-gulopyranoside monohydrate. **13**, methyl *α*-d-gulopyranoside· ½ (CaCl_2_·3H_2_O); **14**, methyl *β*-d-gulopyranoside; **15**, methyl d-*glycero*-*α*-d-*gulo*-heptopyranoside; **16**, methyl d-*glycero*-*α*-d-*gulo*-heptopyranoside· CaC1_2_·H_2_O. **17**, methyl d-*glycero*-*β*-d-*gulo*-heptopyranoside; **18**, methyl d-*glycero*-*β*-d-*gulo*-heptopyranoside ·½CaCl_2_·H_2_O; **19**, cyclohexyl d-*glycero-β*-d-*gulo*-heptopyranoside; **20**, methyl *α*-l-arabinopyranoside. **21**, methyl *β*-l-arabinopyranoside; **22**, methyl *α*-d-galactopyranoside monohydrate; **23**, methyl *β*-d-galactopyranoside; **24**, methyl 6-deoxy-*α*-l-galactopyranos.de.

**Figure 4:**
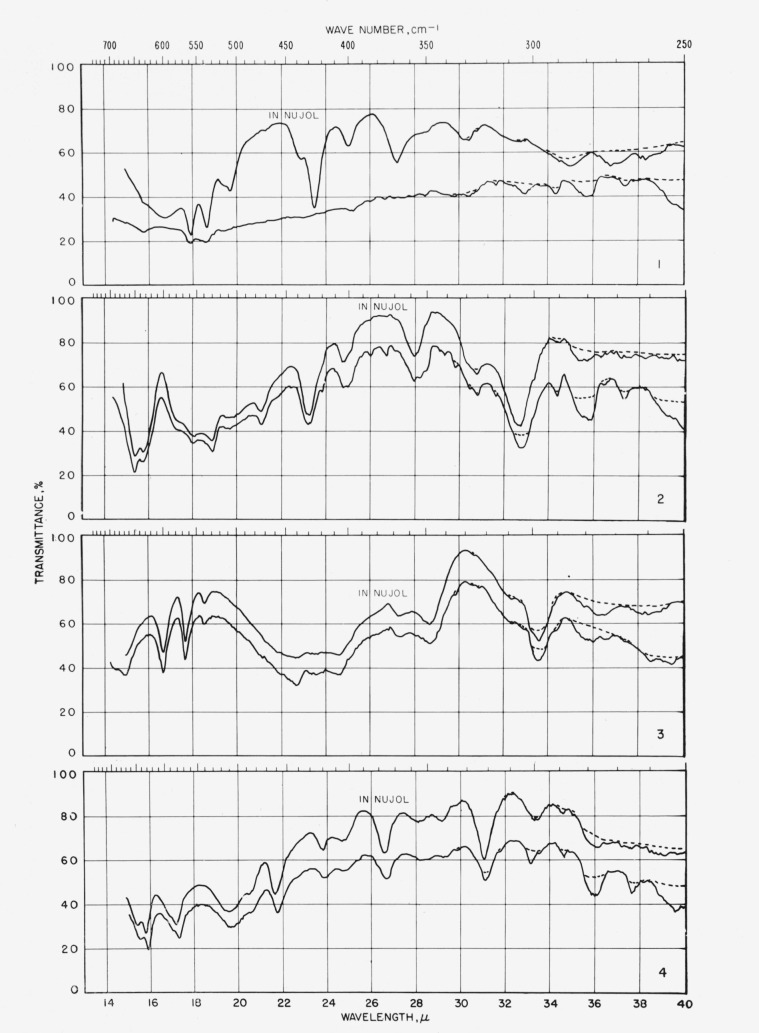

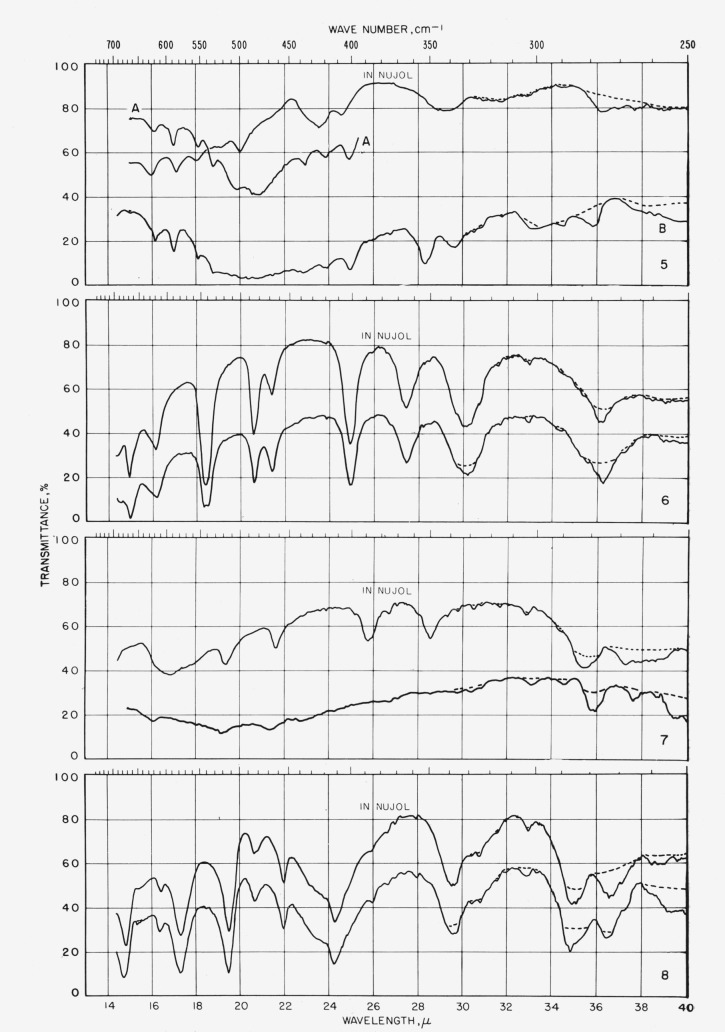

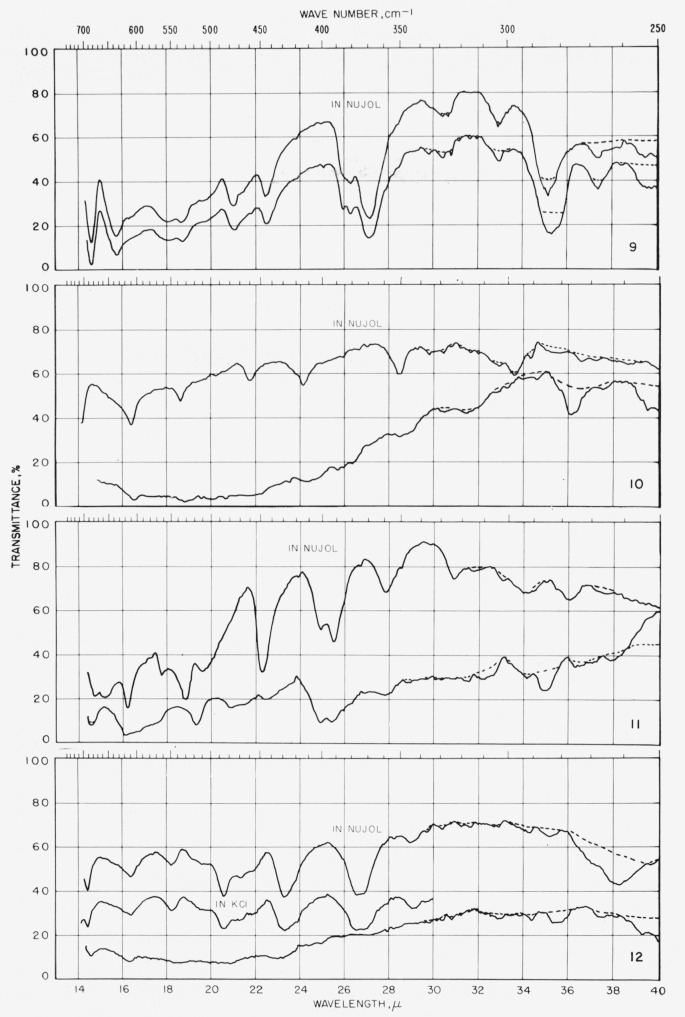

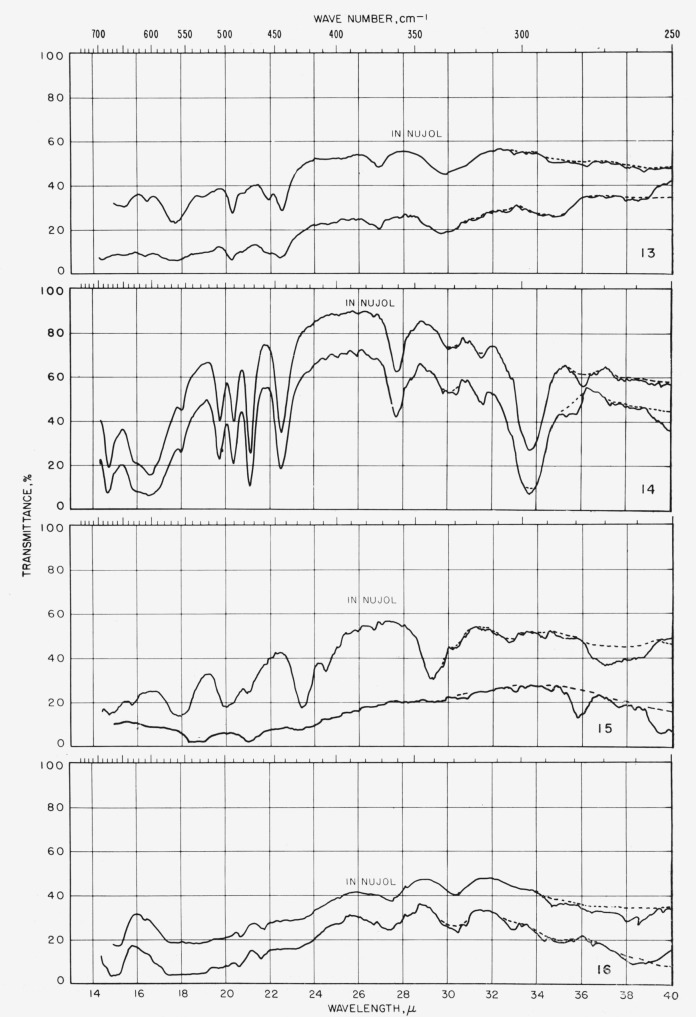

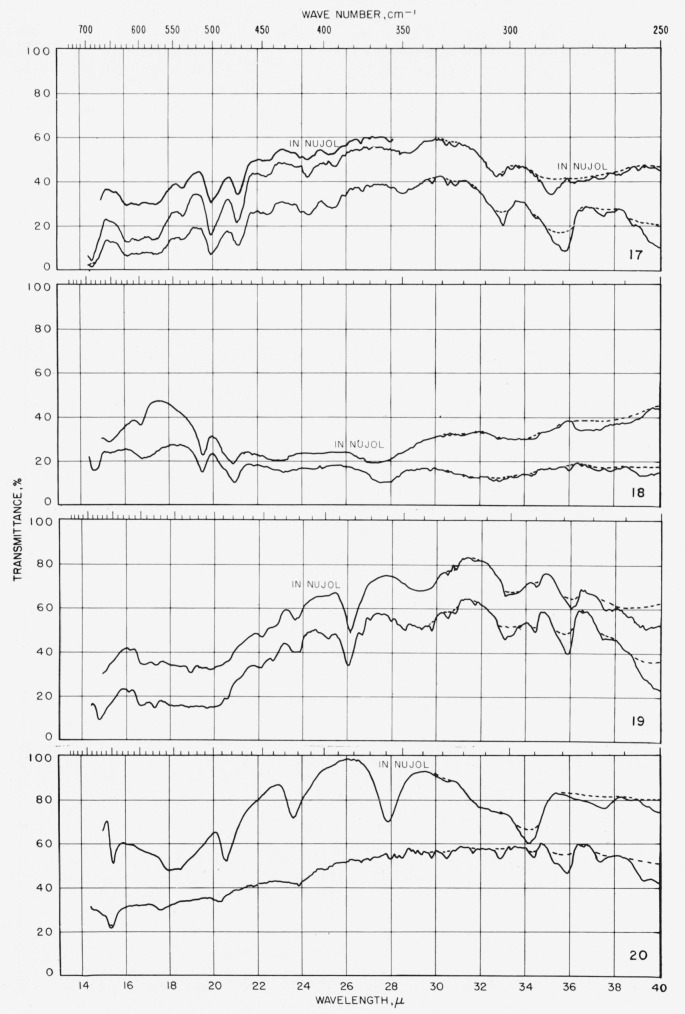

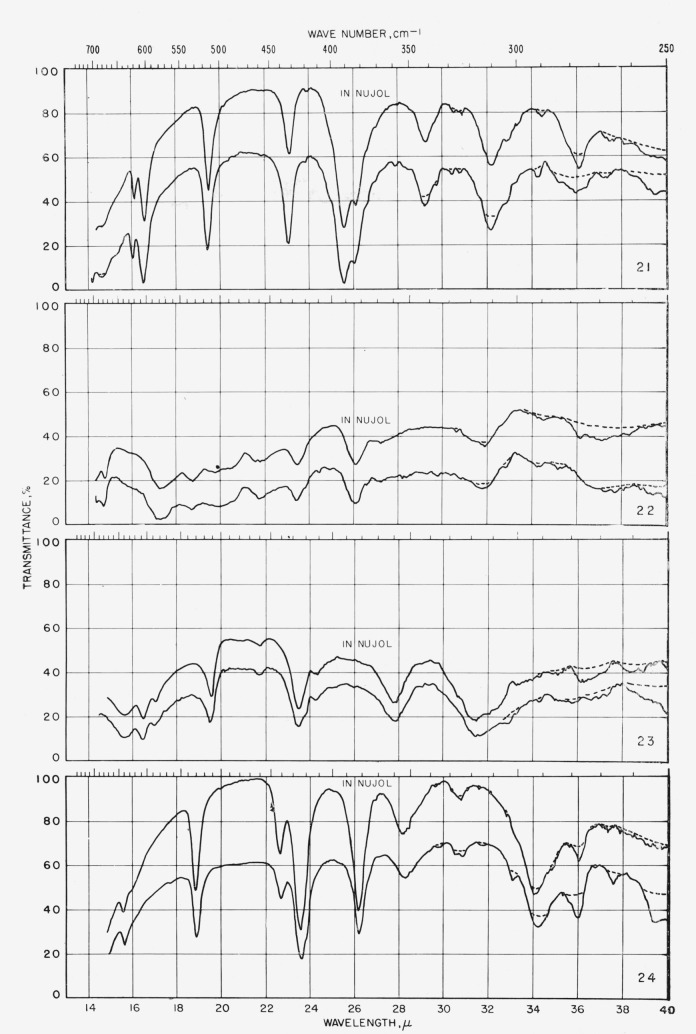
Spectrograms of materials in Nujol mulls and in potassium iodide pellets. **1**, Methyl *α*-d-xylopyranoside; **2**, methyl *β*-d-xylopyranoside; **3**, methyl *α*-d-glucopyranoside; **4**, methyl *β*-d-glucopyranoside. **5**, methyl d-*glycero*-*α*-l-*gulco*-heptopyranoside; **6**, methyl *α*-d-lyxopyranoside; **7**, methyl *β*-d-lyxopyranoside; **8**, methyl *α*-d-mannopyranoside. **9**, methyl 6-deoxy-*α*-l-mannopyranoside; **10**, methyl 6-deoxy-*β*-l-mannopyranoside; **11**, methyl d-*glycero*-*α*-l-*manno*-heptopyranoside; **12**, methyl *α*-d-gulopyranoside monohydrate. **13**, methyl *α*-d-gulopyranoside ·½(CaCl_2_·3H_2_O); **14**, methyl *β*-d-gulopyranoside; **15**, methyl d-*glycero*-*α*-d-*gulo*-heptopyranoside; **16**, methyl d-*glycero*-*α*-d-*gulo*-heptopyranoside ·CaCl_2_·H_2_O. **17**, methyl d-*glycero*-*β*-d-*gulo*-heptopyranoside; **18**, methyl d-*glycero*-*β*-d-*gulo*-heptopyranoside ·½CaCl_2_·H_2_O; **19**, cyclohexyl d-*glycero-β*-d-*gulo*-heptopyranoside; **20**, methyl *α*-l-arabinopyranoside. **21**, methyl *β*-l-arabinopyranoside; **22**, methyl *α*-d-galactopyranoside monohydrate; **23**, methyl *β*-d-galactopyranoside; **24**, methyl 6-deoxy-*α*-l-galactopyranoside.

The spectrograms are shown in figures 3 and 4. They were recorded with a Perkin-Elmer Model 21 (double-beam) spectrophotometer equipped with a prism of sodium chloride (for the range of 5000 to 667 cm^−1^) and of cesium bromide (for the range of 667 to 250 cm^−1^), as previously described [4].

Some absorption attributable to water (in the compound, the alkali halide, or both) was observed at 3448 and 1639 cm^−1^ and, attributable to atmospheric water vapor, in the far-infrared curves. These regions are drawn on the spectrograms with dashed lines which are merely precautionary and are not to be interpreted quantitatively.

### 6.4. Spectra Measured Under Different Conditions

Because of the possibility of interaction of the various compounds with the pelleting halide under high pressure, the spectra of a few of the glycosides, chosen at random, were also recorded in a Nujol mull (requiring no pressure) for comparison. Since a number of these compounds gave markedly different spectrograms in potassium iodide and in Nujol, respectively, the spectra of all of them were now recorded in Nujol. For 16 of the 24 glycosides, the spectra obtained with either medium matched well. However, the following compounds gave spectrograms that were different in Nujol and in potassium iodide: compounds 1, 5, 7, 10, 11, 12, 15, and 20.

Of these, compounds 12 and 15 are known to give a molecular complex with calcium chloride, affording compounds 13 and 16, respectively; both compound 13 and compound 16 give essentially the same spectrum in the two media. This observation might suggest a relationship between the ability to form a complex with calcium chloride and the behavior observed in potassium iodide. However, compound 17, which also forms a complex with calcium chloride (namely, compound 18), gives, like compound 18, essentially the same spectrum in the two media. A possible explanation is that some glycosides may react with both calcium chloride and potassium iodide, whereas others may react with only one of these salts.

It is not known whether compounds 1, 5, 7, 10, 11, and 20 form complexes with calcium chloride, nor whether any of the eight glycosides that give an unsatisfactory spectrum in potassium iodide do actually react chemically with this iodide.

In view of these observations, the spectra obtained with a Nujol mull were used exclusively for measuring the positions of absorption bands in the range of 667 to 250 cm^−1^, not only for the eight glycosides that give unsatisfactory spectra in potassium iodide, but also (in order to keep the measurements strictly comparable) for the 16 other glycosides.

Finally, to make sure that compound 12 (typical of the glycosides “reacting” with both calcium chloride and potassium iodide) does not react with potassium chloride, the spectrum in the range of 667 to 333 cm^−1^ was recorded for a pellet of compound 12 in potassium chloride and compared with its spectrum in Nujol; the spectra matched well. Consequently, the spectra in potassium chloride in the range of 5000 to 667 cm^−1^ were accepted as being satisfactory.

## Figures and Tables

**Table 1:** Compounds measured, stable conformations, and index to spectrograms

Code^a^	Compound	Reference	Stable conformation^b^					Spectrogram
			Reeves’ assignment^c^	Anomeric disposition^c^	Reference	Present assignment^d^	Anomeric disposition^d^	
								
10.11111	Methyl *α*-d-xylopyranoside	1, 2	CA	*a*	13	CA	*a*	1
10.11211	Methyl *β*-d-xylopyranoside	1, 2	CA	*e*	13	CA	*e*	2
10.21111	Methyl *α*-d-glucopyranoside	3	CA	*a*	14	CA	*a*	3
10.21211	Methyl *β*-d-glucopyranoside	3	CA	*e*	14	CA	*e*	4
10.41111	Methyl d-*glycero*-*α*-l-*gluco*-heptopyranoside	4	……………..	……………..	……………..	CA	*a*	5
								
10.12511	Methyl *α*-d-lyxopyranoside	5	CA, CE^e^	*a,e*^e^	13	CA+CE; non-chair.	*a+e*; *a,e*, or *q*	6
10.12511	Methyl *β*-d-lyxopyranoside	4	CA, CE^e^	*e,a*^e^	13	CA+CE; non-chair.	*a*+*e*; *a,e* or *q*	7
10.22111	Methyl *α*-d-mannopyranoside	6	CA	*a*	13	CA	*a*	8
10.22111(6)80	Methyl 6-deoxy-*α*-l-mannopyranoside.	1	CA	*a*	13	CA	*a*	9
10.22211(6)80	Methyl 6-deoxy-*β*-l-mannopyranoside	7	……………..	……………..	……………..	CA	*e*	10
10.42111	Methyl d-*glycero-α*-l-*manno*-heptopyranoside	8	……………..	……………..	……………..	CA	*a*	11
10.2651199	Methyl *α*-d-gulopyranoside, monohydrate	9	CA, CE	*a,e*	13	CA+CE; non-chair.	*a+e; a,e*, or *q*	12
10.26?119899	Methyl *α*-d-gulopyranoside · ½ (CaCl_2_ · 3H_2_O).	9	……………..	……………..	……………..	……………..	……………..	13
10.26211	Methyl *β*-d-gulopyranoside	9	CA	*e*	15	CA	*e*	14
10.36111	Methyl d-*glycero*-*α*-d-*gulo*-heptopyranoside	4	……………..	……………..	……………..	CA	*a*	15
10.36?119899	Methyl d-glycero-*α*-d-*gulo*-heptopyranoside CaCl_2_ · H_2_O.	4	……………..	……………..	……………..	……………..	……………..	16
10.36211	Methyl d-*glycero*-*β*-d-*gulo*-heptopyranoside	1, 4	……………..	……………..	……………..	CA	*e*	17
10.36?119899	Methyl d-*glycero*-*β*-d-*gulo*-heptopyranoside ½ CaCl_2_ · H_2_O.	4	……………..	……………..	……………..	……………..	……………..	18
10.36213	Cyclohexyl d-*glycero*-*β*-d-*gulo*-heptopyranoside	10	……………..	……………..	……………..	CA	*e*	19
								
10.13411	Methyl *α*-l-arabinopyranoside	2	CE	*e*	16	CE	*e*	20
10.13311	Methyl *β*-l-arabinopyranoside	2	CE	*a*	16	CE	*a*	21
10.2311199	Methyl *α*-d-galactopyranoside, monohydrate	1, 11	CA	*a*	16	CA	*a*	22
10.23211	Methyl *β*-d-galactopyranoside	1	CA	*e*	16	CA	*e*	23
10.23111 (6) 80	Methyl 6-deoxy-*α*-l-galactopyranoside	12	CA	*a*	16	CA	*a*	24

a The third figure after the point was inserted after the present conclusions as to conformation had been reached.

b Named by the system of H. S. Isbell and R. S. Tipson, Science **130**, 793 (1959); J. Research NBS **64A**, 171 (1960).

c Assignment made by Reeves [13 to 16] from consideration of instability factors.

d After accepting several of Reeves’ assignments (see text).

e Reeves later suggested [Ann. Rev. Biochem. **27**, 15 (1958)] that the stable conformation may be a member of the boat-skew cycle.

*References for* table 1

1. E. Fischer, Bor. deut. chem. Ges. **28**, 1145 (1895).

2. C. S. Hudson, J. Am. Chem. Soc. **47**, 265 (1925).

3. C. N. Riiber, Ber. deut. chem. Ges. **57**, 1797 (1924).

4. H. S. Isbell and H. L. Frush, J. Research NBS **24**, 125 (1940) RP1274.

5. F. P. Phelps and C. S. Hudson, J. Am. Chem. Soc. **48**, 503 (1926).

6. E. Fischer and L. Beensch, Ber. deut. chem. Ges. **29**, 2927 (1896).

7. E. Fischer, M. Bergmann, and A. Rabe, Ber. deut. chem. Ges. **53**, 2362 (1920).

8. R. M. Hann, A. T. Merrill, and C. S. Hudson, J. Am. Chem. Soc: **57**, 2100 (1935).

9. H. S. Isbell, BS J. Research **8**, 1 (1932) RP396.

10. E. Glaser and N. Zuckermann, Z. physiol. Chem. **166**, 103 (1927).

11. E. Fischer and L. Beensch, Ber. deut. chem. Ges. **27**, 2478 (1894).

12. J. Minsaas, Rec. trav. chim. **51**, 475 (1932).

13. R. E. Reeves, J. Am. Chem. Soc. **72**, 1499 (1950).

14. R. E. Reeves, J. Am. Chem. Soc. **71**, 215 (1949).

15. R. E. Reeves, Advances in Carbohydrate Chem. **6**, 107 (1951).

16. R. E. Reeves, J. Am. Chem. Soc. **71**, 1737 (1949).

**Table 2:** Structural groups studied

Group	Structural feature	Compounds (serial numbers) in group
		
1	Calcium chloride (of crystallization)	13, 16, 18.
2	*arabino; galacto* configuration	20, 21; 22 to 24.
3	*lyxo; manno; gulo* configuration	6, 7: 8 to 11; 12, 14, 15, 17, 19 [13, 16, 18].
4	*xylo; gluco* configuration	1, 2; 3 to 5.
5	Pentopyranoside	1, 2, 6, 7, 20, 21.
6	5-*C*-Methyl (6-deoxy) group	9, 10, 24.
7	Hexopyranoside; 5-*C*-(hydroxymethyl) group.	3, 4, 8, 12, 14, 22, 23 [13].
8	Heptopyranoside; 5-*C*-(l,2-dihydroxyethyl) group.	5, 11, 15, 17, 19 [16, 18].
9	Hydrate	12, 13, 16, 18, 22.
10	Cyclohexyl group, glycosidic	19.
11	Methoxyl group, glycosidic	1 to 12, 14, 15, 17, 20 to 24 [13, 16, 18].
12	Hydroxyl group, primary	3 to 5, 8, 11, 12, 14, 15, 17, 19, 22, 23 [13, 16, 18].
13	Hydroxyl group, secondary	} 1 to 12, 14, 15, 17, 19 to 24 [13, 16, 18].
14	Pyranoid ring	

**Table 3:** Bands (cm^−1^) shown by both anomers of methyl d-xylopyranoside or by both anomers of methyl l-arabinopyranoside (or by all four compounds); and positionally corresponding bands of the methyl d-lyxopyranosides

Methyl d-xylopyranosides		Methyl l-arabinopyranosides		Methyl d-lyxopyranosides	
		
2	1	20	21	6	7

Possibly nonconfigurational bands					

2950	2924	2941	2950	2924	2924
2849	2841	2857	2841	2841	2849
1473	1466	1464	1466	1466	1471
1451	1449	1456	1453	1456	1451
1410	1418	1429	1422	1414	1429
1376	1379	1379	1376	1377	1383
1368	1362	1353	1362	1359, 1355	1353
1277	1269	1266	1269	1274	1280
1242	1247	1258	1245	1245, 1241	1235
1192	1195	1205	1192	1199	1211
1161	1147	1166	1145	1151	1160
1119	1119	1116	1119	1106, 1103	1106
1091	1091	1009	1099	1086	1089
898^a^	a897	877	883	880	885
404(399?)	400	(396?)	391	401	(398?) 389

Bands possibly affected by configuration and conformation					

3390	3390	3378	……………..	3390	3413
2967	……………..	2967	3003	2985, 2959	2967
1344	1342	1332	1342	……………..	1335
1316	……………..	1305	1304	1319, 1311	……………..
1130	……………..	1139	1135	1129	1126
1075	……………..	1085	1079	……………..	1074
1067	……………..	1066	1065	1062	……………..
1044	1042	……………..	1042	……………..	1044, 1034
1015	1007	991	1005	1015	……………..
963	a941	940	944	……………..	950
……………..	……………..	917	923	……………..	……………..
……………..	……………..	779, 770	780	778	775
629	(633?)	……………..	619	616	……………..
(571?)	(595?)	……………..	593		593
551	557	(558?)	……………..	} 541	$\{\;\begin{array}{c} \ldots \ldots \ldots \\ \ldots \ldots \ldots \end{array}$
528	535	(541?)	……………..		
(506?)	(516?) 508	……………..	513	(521?)	517
429	426	425	(419?)	……………..	……………..
……………..	……………..	……………..	383	(386?)	(386?)
……………..	……………..	……………..	(371?)	(376?)	375
358	369(350?)	360	……………..	364 (352?)	351
……………..	……………..	……………..	342	(337?)	(342?)
325	330	……………..	(324?)	332 (325?)	……………..
306	(308?)	(314?)	311 (304?)	(304?)	……………..
……………..	(288?)	293	277	276	283

a These bands were mentioned by S. A. Barker, E. J. Bourne, R. Stephens, and D. H. Whiffen, J. Chem. Soc. **1954**, 3468.

**Table 4:** Bands (cm^−1^) shown by only one anomer of the methyl xylopyranosides and methyl arabinopyranosides, compared with bands for both anomers of methyl lyxopyranoside

Methyl d-xylopyranosides		Methyl l-arabinopyranosides		Methyl d-lyxopyranosides	
		
2	1	20	21	6	7
					
3448	……………..	3460	……………..	……………..	……………..
……………..	……………..	3279	……………..	3289	……………..
3012	……………..	……………..	……………..	……………..	……………..
2874	……………..	……………..	……………..	2882	……………..
1433	……………..	……………..	……………..	1445	……………..
1385	……………..	1395	……………..	……………..	1408, 1383
1295	……………..	1295	……………..	1274	1280
1218	……………..	1227	……………..	……………..	……………..
1060	……………..	1058	……………..	1062	……………..
976	……………..	973	……………..	973	975
645	……………..	646	……………..	……………..	……………..
(496?)	……………..	} 487	$\{\;\begin{array}{c} \ldots \ldots \ldots \\ \ldots \ldots \ldots \end{array}$	485	……………..
473	……………..			467	463
……………..	……………..	……………..	……………..		
……………..	3333	……………..	3322	……………..	3367
……………..	……………..	……………..	3236	3226	……………..
……………..	……………..	……………..	2801	……………..	……………..
……………..	2710	……………..	2695	2717, 2667	2703
……………..	……………..	……………..	845, 838	848	865
……………..	a741	……………..	744	744	……………..
……………..	……………..	……………..	713, 699	685	697
……………..	……………..	……………..	678	664	……………..
……………..	437	……………..	433	……………..	……………..

a See footnote to table 3.

**Table 5:** Comparison^a^ of absorption bands (cm^−l^) shown by the methyl d-galactopyranosides (22 and 23) and by methyl 6-deoxy-α-l-galactopyranoside (24)

A			B		C	D	
			
22	23	24	22	23	23	22	24
							
3390	3356	3367	3521	3559	3300	2924	2915
3236	3257	3247	2882	2899	1285	2646	2710
2959	2941	2950	1316	1321	1250	1462	1464
2857(?)	2865	2841	[1274	1261]	981	1355	1348
1449	1445	1433	b1222	b1220	c888	1242	1245
1414	1429	1416	1149	1144	412	1138	1136
1403	1408, 1397	1391	575	585		1074	1079
1372	1383	1364	(505?)	510		1015	1005
1339	1333	1330				964	963
1305	1295	1300				c 822	810
1259	1261	1259				673	681
1196^b^	1182	d 1196				532	530
1160	1155	1161				382	382
1120	1124	1120					
1094	1107	1100					
1080^b^	b1082	d1086					
1064	b1057	d1049					
1034^b^	1033	1026					
922^c^	c938	916					
868^c^	c870	867					
787^c^	c784	766					
706	727	717, 709					
(629?)	637	639					
458	460	442					
426	426	424					
(367?)	360	355					
314	318	325					
(297?)	(301?)	293					

a Key: A. Bands shown by both anomers of methyl d-galactopyranoside and by methyl 6-deoxy-*α*-l-galactopyranoside. B. Bands shown by both anomers of methyl d-galactopyranoside, but not by compound 24. C. Bands shown by methyl *β*-d-galactopyranoside, but not by methyl *α*-d-galactopyranoside or methyl 6-deoxy-*α*-l-galactopyranoside. D. Bands shown by methyl *α*-d-galactopyranoside and by methyl 6-deoxy-*α*-l-galactopyranoside, but not by compound 23.

b These bands were mentioned by R. L. Whistler and L. R. House, Anal. Chem. **25**, 1463 (1953).

c See footnote *a* to table 3.

d These bands were mentioned (see footnote *b*) for the *α*-d form.

**Table 6:** Comparison^a^ of the absorption bands (cm^−1^) shown by the anomers of methyl d-gulopyranoside (12 and 14) and by methyl α-d-mannopyranoside (8)

A			B		C	D		E	
				
8	12	14	12	14	12	8	12	8	14
									
3460	3484	3413	3257	3247	3195	2841	2849	1389	1385
3289	3333	3322	2924	2915	1484	1372	1372	1350	1350
3012	3030	2976	2725	2762	1437	1361	1361	1258	1258
2950	2950	2941	1302	1305	1144	(420?)	(421?)	1041	1036
2907	2907	2915	1289	1289	1133	412	(416?)	b916	913
2841	2849	2865	1276	1272		(386?)	378	b848	855
1473	1471	1473	1087	1087				(645?)	(656?)
1451	1451	1449	1018	1016				(329?)	318
1414	1418	1418	468	473				(304?)	296
1399	1406	1393	(360?)	360				286	(277?)
1330	1333	1337	(354?)	(352?)					
1312	1318	1316							
1250	1250	1258, 1238							
1220^c^	1225	1220							
1199^c^	1193	1205							
1163	1159	1151							
1119	1121	1126							
1104	1096	1105							
1071^c^	1078, 1070	1070							
1055^c^	1053	1056							
1029	1029	1026							
973^b^	968	994							
890^b^	873, 872	903							
817^b^	820	798							
723	739	751							
672	687	675							
608	608	601							
576	548	553							
512	(506?)	506							
483	486	490							
455	458	444							
338	345	333							

a Key: A. Bands shown by methyl *α*-d-mannopyranoside and by both anomers of methyl d-gulopyranoside. B. Bands shown by the methyl d-gulopyranosides, but not by compound 8. C. Bands shown by methyl *α*-d-gulopyranoside, but not by compounds 8 or 14. D. Bands shown by methyl *α*-d-mannopyranoside and by methyl *α*-d-gulopyranoside, but not by compound 14. E. Bands shown by methyl *α*-d-mannopyranoside and by methyl *β*-d-gulopyranoside, but not by compound 12.

b See footnote *a* to table 3.

c See footnote *b* to table 5.

**Table 7:** Comparison^a^ of absorption bands (cm^−1^) shown by the anomers of methyl d-glucopyranoside (3 and 4) and by methyl d-glycero-α-l-gluco-heptopyranoside (5)

A			B		C	
		
3	5	4	3	4	3	5
						
2915	2941	2959	3279	3247	1340	1339
2849	2857	2857	2915	2890	1202	1214
1464	1466	1466	2667(?)	2793(?), 2710	1114	1111
1443	1449	1447	1323	1326	845	864
1401	1410	1403	1135	1129	747	745
1376	1381	1383	(398?)	377	564	553
1366	1353	1364	367	356		
1305	1302	1304	298	300		
1267	1269	1285				
1229	1236	1248				
1229^b^	1214	b1222				
1188^b^	1193	b1186				
1159	1153	1161				
1125	1120	1125				
1104	} 1087	$\{\;\begin{array}{c} 1098\\ {\;}^{b}1081,1073 \end{array}$				
1075						
1053	1053	b1054				
1047^b^	1040	1037				
1034	1015	1030				
995	997	1002, 993				
899^c^	892	c885				
794(?)	787	c781				
627(?)	627	631				
598	584	(591?) 580				
539	522	510				
(490?)	501	488				
(442?)	426	420				
(407?)	408	406				
349	344	343				

a Key: A. Bands shown by the anomers of methyl d-glucopyranoside and by methyl d-*glycero-α*-l-*gluco*-hoptopyrdnoside. B. Bands shown by the anomers of methyl d-glucopyranoside, but not by compound 5. C. Bands shown by methyl *a*-d-glucopyranoside and by methyl d-*glycero*-*α*-l-*gluco*-heptopyranoside, but not by compound 4.

b See footnote *b* to table 5.

c See footnote *a* to table 3.

**Table 8:** Comparison^a^ of absorption bands (cm^−1^) shown by methyl α-d-mannopyranoside (8) and by both anomers of methyl 6-deoxy-l-mannopyranoside (9 and 10)

A			B		C		D	
			
8	9	10	9	10	8	10	8	9
3012	2985	2976	3559	3509	3289	3333	2907	2890
2950	2924	2941	3413	3401	1163(?)	1174	1389	1389
2841	2857	2857	1323	1325	723	708	1330	1333
1473	1473	1466	992	1004			1199	1206
1451	1456, 1447	1447	963	959			1119	1114
1414	1420, 1412	1416	534	537			b1055	1058
1372	1376, 1370	1374					973	983
1350	1351	1350					c848	c838
1330	1333, 1323	1325					(645?)	632
1312	1305	1300					576	552
1258	1256	1259					512	(509?)
1220^b^	1220	1224, 1218					(329?)	328
1199^b^	1206	1174					286	284
1144	1145	1149						
1133	1125	1127						
1104	1098	1096						
1071^b^	1083	1088, 1072						
1029	1024	1024						
973^c^	c963	959						
916^c^	c909	903						
890^c^	c877	870						
817^c^	c803	801, 798						
672	677	699						
608	(610?)	609						
512	534	537						
483	475	(495?)						
455	(467?)444	460						
(420?)412	(420?)	414						
(386?)	380, 368	(376?)						
338	(354?)	352						
(304?)	303	298						

a Key: A. Bands shown by methyl *α*-d-mannopyranoside and by both anomers of methyl 6-deoxy-l-mannopyranoside. B. Bands shown by both anomers of methyl 6-deoxy-l-mannopyranoside, but not by compound 8. C. Bands shown by methyl *α*-d-mannopyranoside and by methyl 6-deoxy-*β*-l-mannopyranoside, but not by compound 9. D. Bands shown by methyl *α*-d-mannopyranoside and by methyl 6-deoxy-*α*-l-mannopyranoside, but not by compound 10.

b See footnote *b* to table 5.

c See footnote *a* to table 3.

**Table 9:** Comparison^a^ of absorption bands (cm^−1^) shown by methyl α-d-mannopyranoside (8), the anomers of methyl 6-deoxy-l-mannopyranoside (9 and 10), and methyl d-glycevo-α-l-manno-heptopyranoside (11)

A		B		
	
10	11	8	9	11
3333	3322	3460	……………..	3425
2710	2703(?)	2907	2890	2915
1287	1285	1399, 1389	1389	1395
		1361	……………..	1368
		……………..	1342	1340
		1250	……………..	1241
		b1199	1206	1200
		1119	1114	1115
		b1055	1058	1048
		1041	……………..	1036
		c973	983	982
		c848	c838	827
		(645?)	632	654
		576	552	(578?)562
		512	(509?)	509
		(329?)	328	324
		286	284	277

a Key: A. Bands shown by compounds 10 and 11, but not by compounds 8 and 9. B. Bands shown by compounds 8, 9, and 11, but not by compound 10.

b See footnote *b* to table 5.

c See footnote *a* to table 3.

**Table 10:** Comparison^a^ of absorption bands (cm^−1^) shown by methyl β-d-gulopyranoside (14) and by the anomers of methyl d-glycero-d-gulo-heptopyranoside (15 and 17)

A			B		C	
		
14	15	17	15	17	14	17
3413	3401	3448, 3378	948	944	3322	3333
2941	2933	2950	884	880	2762	2732
2865	2849	2882	408	412	1418	1425
1473	1460	1462	(399?)	393	1393	1408
1449	1447	1456			1337	1330
1418	} 1412	$\left\{ \begin{array}{l} 1425\\ 1408 \end{array}\right.$			1316	1311
1393					1289	1287
1385	1374	1372			1238	1238
1350	1346	1357			1220	1212
1258	1264	1253			1126	1122
1205	1192	1203			1070	1075, 1064
1151	1153	1151			1056	1056
1105	1106	1099			1026	1025
1087	1089	1082			1016	1006
1036	1042	1036			903	907
994	985	990			798	801
885	844	847			601	599
675	676	683				
(627?)	633	617				
553	559	575, 537				
506	500	502				
473	477	474				
444	(459?) 426	447				
360	(375?)	(359?)				
(352?) 333	342	348				
296	(306?)	306				

a Key: A. Bands shown by methyl *β*-d-gulopyranoside (compound 14) and by both anomers of methyl d-*glycero*-d-*gulo*-heptopyranoside. B. Bands shown by both anomers of methyl d-*glycero*-d-*gulo*-heptopyranoside, but not by compound 14. C. Bands shown by methyl *β*-d-gulopyranoside and by methyl d-*glycero*-*β*-d-*gulo*-heptopyranoside, but not by compound 15.

## Footnotes

1 Figures in brackets indicate the literature references at the end of this paper. The references for table 1 are given at the end of the table.

2 The method of comparison is being applied to other groups of structurally related pyranoid sugar derivatives.

3 Code numbers are assigned according to a previously published classification system [5], for use with punched-card techniques.

4 For comparison, bands (in about the same positions) shown by either or both anomers of methyl d-lyxopyranoside are also given in table 3.
